# Charge Sharing in (CdZn)Te Pixel Detector Characterized by Laser-Induced Transient Currents

**DOI:** 10.3390/s20010085

**Published:** 2019-12-22

**Authors:** Igor Vasylchenko, Roman Grill, Eduard Belas, Petr Praus, Artem Musiienko

**Affiliations:** Institute of Physics, Faculty of Mathematics and Physics, Charles University, Ke Karlovu 5, CZ-12116 Prague 2, Czech Republic; grill@karlov.mff.cuni.cz (R.G.); eduard.belas@mff.cuni.cz (E.B.); praus@karlov.mff.cuni.cz (P.P.); musienko.art@gmail.com (A.M.)

**Keywords:** (CdZn)Te, transient current, charge sharing, small pixel effect

## Abstract

Performance of the (CdZn)Te pixelated detectors heavily relies on the quality of the underlying material. Modern laser-induced transient current technique addresses this problem as a convenient tool for characterizing the associated charge distribution. In this paper, we investigated the charge sharing phenomenon in (CdZn)Te pixel detector as a function of the charge collected on adjacent pixels. The current transients were generated in the defined 4 mm^2^ spots using 660 nm laser illumination. Waveforms measured on the pixel of interest and its surroundings were used to build the maps of the collected charge at different biases. The detailed study of the maps allowed us to distinguish the charge sharing region, the region with a defect, and the finest part in terms of the performance part of the pixelated anode. We observed the principal inhomogeneity complicating the assignment of the illuminated spot to the nearest pixel.

## 1. Introduction

(CdZn)Te radiation detectors are used in a large variety of gamma and X-ray applications: nuclear safeguards [1,2], high energy astronomy telescopes [3,4], medical X-ray imaging [5], etc. That is because they surpass other semiconductor materials due to excellent properties enabling room-temperature spectroscopic detection. These are the wide band gap associated with low leakage current and excellent electron mobility-lifetime (*μτ*) product [2,6].

Despite evident advantages, (CdZn)Te suffers from several drawbacks, one of them being a relatively large difference between the transport properties of electrons and holes [7,8]. Low hole mobility and enhanced hole trapping reduce the charge collection efficiency (CCE) and produce long asymmetric tail at measured energy spectra (hole tailing). Thus, poor hole mobility-lifetime product (*μ_h_τ_h_*) and relevant defect structure [7,9] are critical issues in the utilization of (CdZn)Te radiation detectors.

In order to reduce the detrimental effect of the low *μ_h_τ_h_*, detectors can be designed in a particular way. Most of the planar detectors use the classical planar parallel field configuration. The design involves the cathode illumination, where the hole impact is minimized in case of low energy X-rays due to the appreciable attenuation coefficient and the higher probability of generating electron-hole pairs close to the cathode. The respective signal can be further refined based on the electronic processing of signals (pulse rise time discrimination [10], bi-parametric analysis [11], etc.). This method prohibits the processing of preamplifier pulses corresponding to holes by comparing pulse height to its rise time. Modern unipolar detectors, i.e., single charge carrier sensors (Frisch and coplanar grids, strip, pixel, hemispherical, and multiple electrodes detectors) [6,12], use dominant electron collection of the charge carriers as well. Previous studies [12] showed that pixelated (CdZn)Te detector can also benefit from particular design if wired in a form of a virtual coplanar grid. These detectors are featured by specific electrode configurations that in general provide a particular distribution of electric field inside the sample. According to Shockley–Ramo theorem [13], these field effects induce signal as follows:

(1) $$I(t)=qE_{w}[x(t)]v[x(t)],$$

where *I*—induced current, *t*—time, *q*—moving charge, *E_w_*—weighting field, *x*(*t*)*—*particle position, *v*—its instantaneous velocity at the position *x*(*t*). In unipolar detectors, the charge carriers drifting to the anode (electrons) induce a much higher signal than those moving to the cathode (holes). In pixelated detectors, this phenomenon is also known as a “small pixel effect” [14]. Modern pixel and strip designs differ from other unipolar detectors, as they not only detect high energy particles, but also provide their pinpoint tracking and thus enable high-resolution 3D imaging [15]. In particular, in pixel detectors the particle energy and its position are determined by measuring and comparing the charge collection on individual anodes (pixels). In theory, the amount of collected charge *Q* explicitly depends on the transient current:

(2) $$Q=\int_{0}^{t_{c}}I(t)dt,$$

where *t_c_* is the collection time at which charge carriers reach respective electrodes, and the signal drops to zero, *I*(*t*)—transient current dependent on time *t*. Let us note that due to the diffusion broadening and the slowing down the drifting carriers by trapping/detrapping on shallow levels in real detectors, the *t_c_* must be appropriately prolonged to collect also delayed charge satisfactorily.

**Figure 1:**
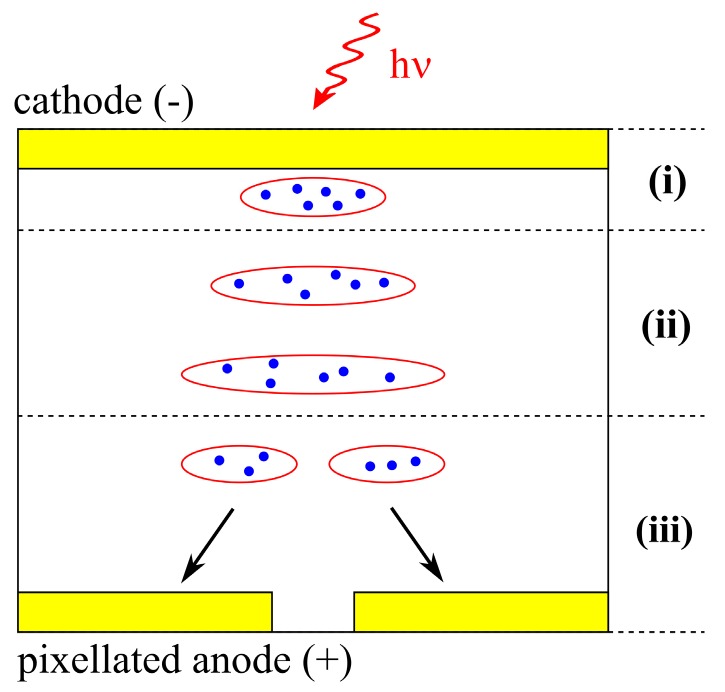
Scheme of the formation and propagation of the electron cloud in the detector illustrating the charge sharing effect.

Generally, based on the location of the detected event, the signals may be split on multiple sites so that the charge collected on each pixel is lower than the total one. Once no appropriate correction was carried out, the spectral and space detection deterioration would have appeared [16], since the signal would be evaluated as the simultaneous detection of individual species of lower energies hitting different spots. This phenomenon is known as the charge sharing effect. Its probability is magnified due to the fact that an initially generated cloud of charge carriers grows in size at the drift to the anode as it is sketched in Figure 1. The radiation is absorbed in the detector’s bulk, creating a cloud of electrons near the cathode—region (i). Holes are neglected for simplicity. The cloud drifts through the region (ii), growing in size due to the diffusion and charge repulsion. Finally, the cloud splits into several parts, and it is collected by adjacent pixels (region (iii)). Iniewski et al. [17] modeled this situation using diffusion and charge repulsion mechanisms. Giraldo et al. [18] studied the charge sharing phenomenon in order to enhance the spatial resolution of the (CdZn)Te pixel detector by means of transient charge technique (TChT). In general, the charge sharing has also been studied previously analytically and numerically [18,19,20], yet the exact dependence of signal formation on charge sharing is still questionable.

Recently, the transient current technique (TCT) became a standard method of measuring induced signal and characterizing the charge transport in bulk [19,20] and thin films [21]. Laser-induced TCT (L-TCT) [22,23] represents a convenient option between TCT techniques, as it affords additional advantages represented by the possibility to define properties of the light pulse like wavelength, intensity, repetition frequency, time and space extension, and correlate the excitation pulse with the onset of the biasing. Simultaneously, the illumination source may be synchronized with a measuring device through the oscilloscope triggering, which leads to significant noise suppression.

In our work, we investigated the relative distribution of the collected charge in a commercially available Redlen pixel detector by comparing the L-TCT signal on the pixel of interest and its surrounding. We show how the position of the illuminated spot affects the current waveform shape. We also use the charge collection to distinguish charge sharing and defective regions producing irregularities at the current transients. We conclude that the detailed analysis of the L-TCT signal can be used to improve the spatial resolution of the detector and to make necessary corrections to its function.

## 2. Experimental Setup

**Figure 2:**
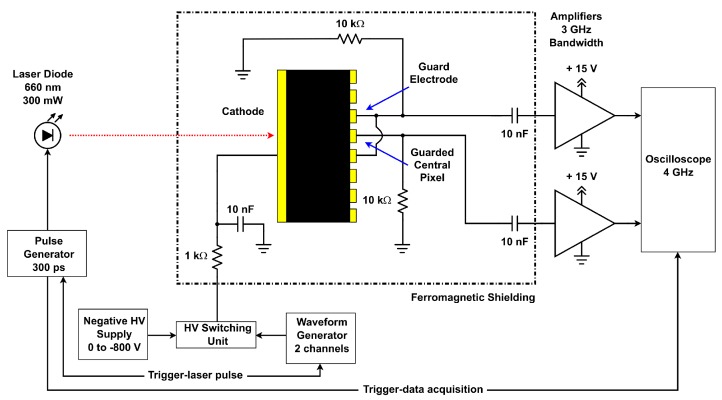
Block diagram of L-TCT setup for measuring transient currents.

The block diagram of the L-TCT setup for measuring the transient currents is shown in Figure 2. A laser diode (660 nm wavelength, 300 mW power) driven by an ultrafast pulse generator (300 ps rise time) emits short optical pulses (500 ps halfwidth, 100 Hz repetition frequency). 660 nm laser wavelength corresponds to the photon energy higher than the (CdZn)Te band gap. Therefore, the red light is absorbed right under the cathode surface, where it generates electron-hole pairs. Light is focused through an aperture in the ferromagnetic shielding by adjustable collimation optics onto ≈4 mm^2^ examined spot on the cathode. The spot area is comparable to the size of pixelated anodes (2.25 × 2.25 mm^2^). Pulse generator also triggers a fast digital sampling oscilloscope (40 Gs/s, resolution up to 11 bits, 4 GHz bandwidth), which records the current waveform so that data acquisition is synchronized with the excitation pulse.

**Figure 3:**
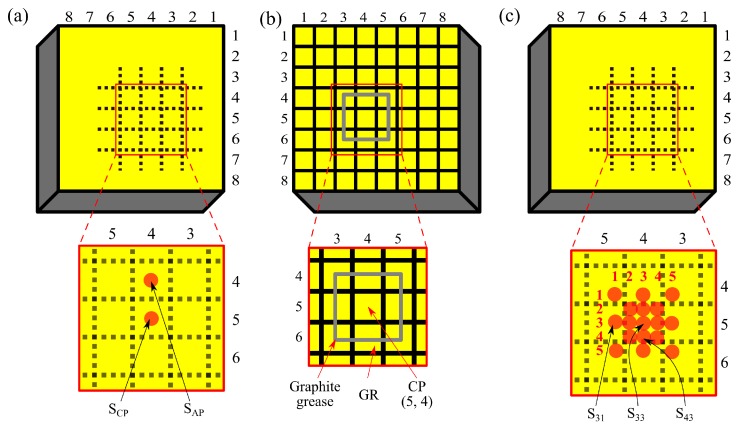
Detector configuration for the L-TCT measurements: (**a**) Cathode view with illuminated spots used in the first experiment; (**b**) anode view with central pixel (CP) and guard ring (GR) configuration; (**c**) cathode view with illuminated spots used in other experiments.

Experiments were carried on commercially available semi-insulating n-type Redlen pixel detector with dimensions: 20 × 20 × 5 mm^3^, 8 × 8 pixel array anode with 2.55 mm pixel pitch and pixel sizes 2.25 × 2.25 mm^2^. The electron mobility-lifetime product (*µτ*) of the tested material was 7 × 10^−3^ cm^2^/V. We conducted the measurements in two configurations. In the first experiment, transient currents were measured on the single pixelated anode denoted as “central pixel” (CP) while illuminating two spots on the cathode, which are shown in Figure 3a. These spots refer to the CP projection (S_CP_) and the projection of the adjacent (4, 4) pixel (S_AP_). In other experiments, transients were measured simultaneously on two grounded anodes (see Figure 3b): on the CP and on the guard ring (GR) made by interconnecting eight pixels by graphite paste around the CP. Figure 3c illustrates the part of the cathode, which was illuminated during measurements on multiple anodes. Dashed squares represent the projections of pixelated anodes, and numerated red circles mark the centers of illuminated spots. Beam position at S_CP_ (also denoted as S_33_) was calibrated with ±0.25 mm accuracy using a paper grid attached to the cathode. Other spots were set using a stepper motor with ±0.0005 mm degree of precision. Their exact positions are presented in Table 1. The actual area of a light circle is comparable to the size of an anode pixel (2.25 × 2.25 mm^2^). In all cases, the negative voltage (up to 800 V), hereinafter referred to as bias, was applied between the cathode and contacted pixels—CP and GR.

## 3. Results and Discussion

### 3.1. Unguarded Pixel Measurement

**Figure 4:**
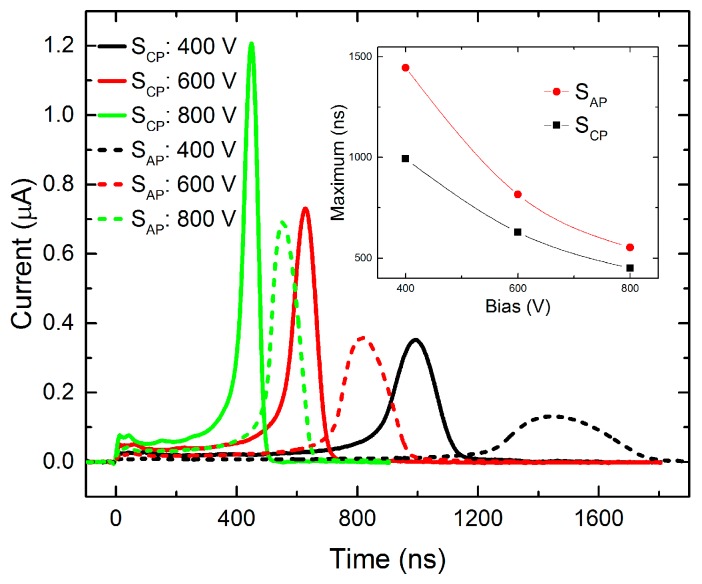
Bias dependence of current waveforms measured on a single CP anode when illuminating S_CP_ or S_AP_. Inset shows the bias dependence of the time of maximum current after illuminating given spots.

In the first experiment, the L-TCT current transients were measured on the CP anode biased at 400–800 V, and other pixels remained unwired to the voltage source. The signal was induced by illuminating S_CP_ or S_AP_ spots (see Figure 3a). In the case of above-band-gap excitation, charge carriers are generated right under the cathode, and electrons move towards the anode under the influence of the electric field. They induce “transient currents” shown in Figure 4, which can be evaluated within the Shockley–Ramo theorem [13] (Equation (1)). When all electrons reach the anode, the current drops to zero. Holes reach the cathode almost immediately after excitation, and they have no impact on the signal.

As we may see in Figure 4, the most striking feature of the current waveforms is the rise in the time before the cloud of photoexcited electrons reaches the anode. This phenomenon corresponds to the pseudo-hemispherical configuration of the detector [24], i.e., its anode (pixel) has a smaller area then cathode. Thus, a nonhomogeneous electric field and space-dependent weighting potential define the charge movement. In particular, the rapid current growth occurs when electric field lines start to bunch in the proximity to the smaller electrode (pixel). At this setup, the charge dynamics cannot be characterized by the constant drift velocity as the electric field is non-uniform.

In our experiment, the signal was induced only by electrons moving to the CP pixel. Their propagation can be roughly estimated as a constant movement at the drift velocity, followed by acceleration near the anode. This acceleration causes the non-linear increase of the current at the graphs. However, the signal is given by the product of the drift velocity and the weighting field, which is also being changed in the non-uniform electric field. Therefore, the carrier mobility cannot be easily obtained from current waveforms unless exact weighting potential corresponding to the pixelated anode geometry is defined. Since the determination of electron drift mobility was not of primary focus of this paper, we do not endeavor after this achievement.

Regardless of the unknown weighting field, we may notice in the inset of Figure 4 that the maximum at the lowest bias 400 V being *τ* ≈ 1000 ns (S_CP_) is more extended in comparison to *τ* determined at larger bias than it should be expected taking linear scaling of the transients with bias. We thus deduce that a negative space charge is formed near the blocking anode, inducing detector polarization. This space charge participates at the electric field warping, amplification of the current transient at the terminal state, and delays the charge transit through the detector, especially at the low bias [25].

One can see that current maxima, which correspond to the transit time, are shifted towards a shorter time as applied voltage and respective electrical field is increased. Additionally, if charge carriers are generated further from S_CP_, the peak is shifted in time due to an extended path and longer transit time. The tail observed at each current waveform is caused by the diffusion broadening of the charge cloud and by a finite dimension of the illuminated spot. The latter fact corresponds to different pathways passed by carriers excited in distinct points in the spot.

### 3.2. Guarded Pixel Measurement

**Figure 5:**
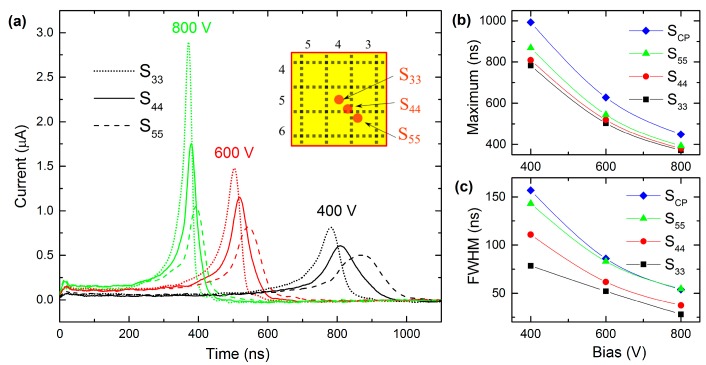
Bias dependence of (**a**) total current waveforms measured by illuminating S_33_, S_44_, and S_55_ spots, (**b**) waveform maxima, and (**c**) peak full width at half maximum (FWHM). Lines S_CP_ show results evaluated in unguarded pixel measurements defined in Section 3.1.

In the experiments with the guarded pixel, we measured transient currents alternatively on two separate anodes (CP and GR), which are shown in detail in Figure 3b. Signals were induced by illuminating spots shown in Figure 3c. For each spot, we obtained the dependence of the waveforms on the applied bias (400 V to 800 V). Figure 5a plots the dependence of the total current transient (obtained by summing of the data measured separately on CP and GR) on the position of the illuminated spot (S_33_—dotted plots, S_44_—line plots, S_55_—dashed plots) for different biases (400 V to 800 V).

**Figure 6:**
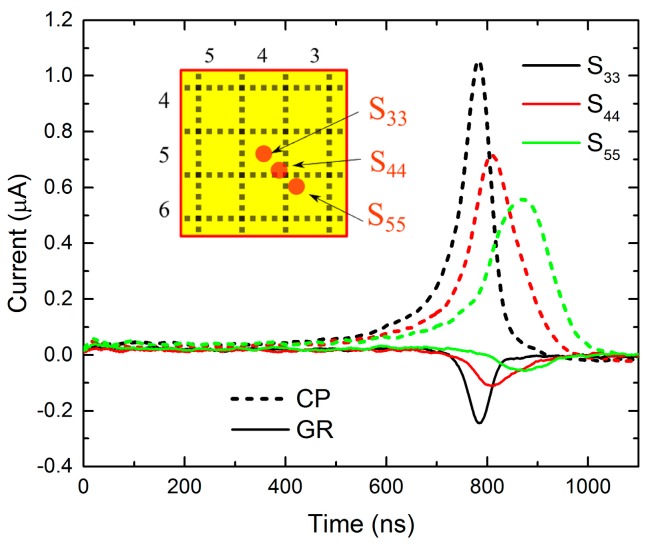
Individual current waveforms measured on CP and GR when illuminating different spots at 400 V bias.

As in the previous single pixel measurements, the accelerated drift of charge carriers in the non-uniform electric field causes the rise of the current in time in all cases. The bias dependence of the waveform maxima, which is plotted in Figure 5b, has non-linear behavior, which means that the negative space charge formation is present in guarded pixel measurements as well. It is evident that the waveforms “reach” their maxima earlier when the charge carriers are generated closer to the S_33_ spot; also, full width at half maximum (FWHM) data in Figure 5c shows that guarded pixel curves are stepper in comparison to unguarded one. These phenomena can be explained by charge carriers passing different pathways and by the enhancing effect of the guard ring. The latter is illustrated in Figure 6, which shows currents measured at 400 V bias while illuminating S_33_, S_44,_ and S_55_. It can be seen that CP waveforms (scatter plots) have the same properties as in the previous cases: They rise in time. However, GR curves (line plots) change the sign and reach negative values when electrons approach the anode. The phenomenon corresponds to the non-collecting behavior of GR, i.e., current induced on the non-collecting electrode during the drift of electrons from the cathode is withdrawn from GR when it is terminally collected on CP. We reckon the redistribution of the collected charge induced in single pixels during the charge drift through the detector into the pixel(s) where the charge finally terminates for the most striking feature of the small pixel effect in pixelated detectors. Adjacent pixels forming the GR electrode counteract in such a way that their influence on the charge carriers moving directly to CP is negligible.

**Figure 7:**
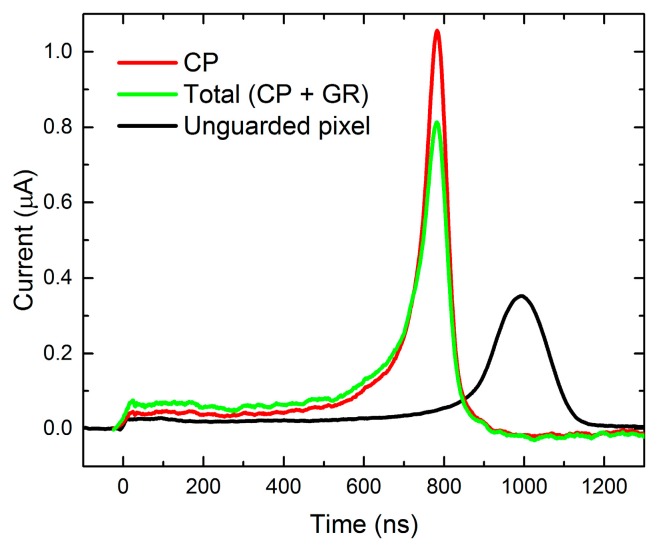
Current waveforms measured on different electrodes at S_33_ illumination with respective values of collected charge and transit time shown in the inset.

As it is seen in Figure 7, due to the non-collecting behavior of GR, the total current waveform reaches a lower maximum than the individual CP waveform. However, the collected charge values, which were calculated from Equation (2), are similar for CP (126 fC) and CP + GR (127 fC), and significantly lower for the unguarded electrode (82 fC). This feature is the direct entailment of the small pixel effect. Therefore the unguarded pixel curve exhibits a longer transition time, which is related to the peak position, and lower peak and collected charge value, i.e., the detector is more prone to the charge trapping and the dark current noise.

**Figure 8:**
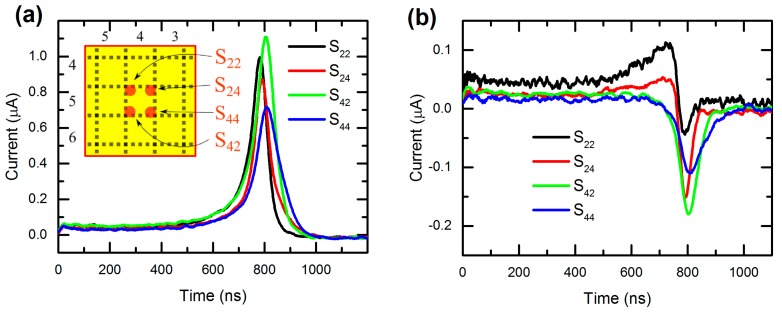
Individual current waveforms measured on (**a**) CP and (**b**) GR when illuminating symmetrical spots at 400 V bias.

At the effort to check the possibility of subpixel resolution of the examined detector, we investigated the course of current waveforms in several spots around CP. Figure 8 shows currents measured when illuminating spots near the border of CP (S_22_, S_24_, S_42_, and S_44_) with S_44_ being the closest to both crystal edges. However, hereinafter we neglect the influence of the detector geometry since charge excitation is done at least 5 mm far from edges (at 5 mm crystal thickness), and consider that these spots are symmetrical around CP.

Taking into account all the above, waveforms measured on CP (Figure 8a) are expected to be similar. However, this is not the case, possibly due to local contact or crystal inhomogeneity. In fact, we revealed up to 57% difference in the peak height (~0.7 μA for S_44_ vs. ~1.1 μA for S_42_) and up to 3% difference in the peak position (775 ns for S_22_ vs. 800 ns for S_44_). Likewise, currents on GR (Figure 8b) have the same distribution by the valley position, but a different depth order of the valley showing significant differences (~−0.05 μA for S_22_ vs. ~−0.175 μA for S_42_). Evidently, observed irregularity disables straightforward assignment of the signal to the illuminated spot taking only the detector geometry into account. A more detailed inspection of the charge propagation and inter-pixel interplay is thus necessary to reconstruct the original spatial distribution of the excitation with subpixel resolution.

### 3.3. Charge Collection

In the previous section, we discussed waveform properties (peak position, peak height, and shape), which afforded us information on the charge drift through the detector. In this section, we concentrate the effort on the investigation of the collected charge representing the most important quantity characterizing the detector quality and the pixel resolution.

**Figure 9:**
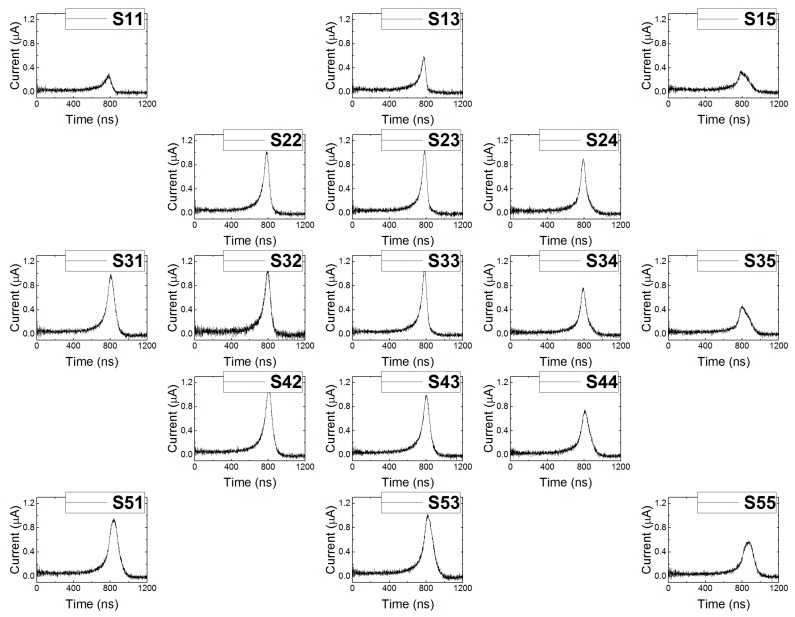
Current waveforms measured on CP at 400 V bias while illuminating different spots.

**Figure 10:**
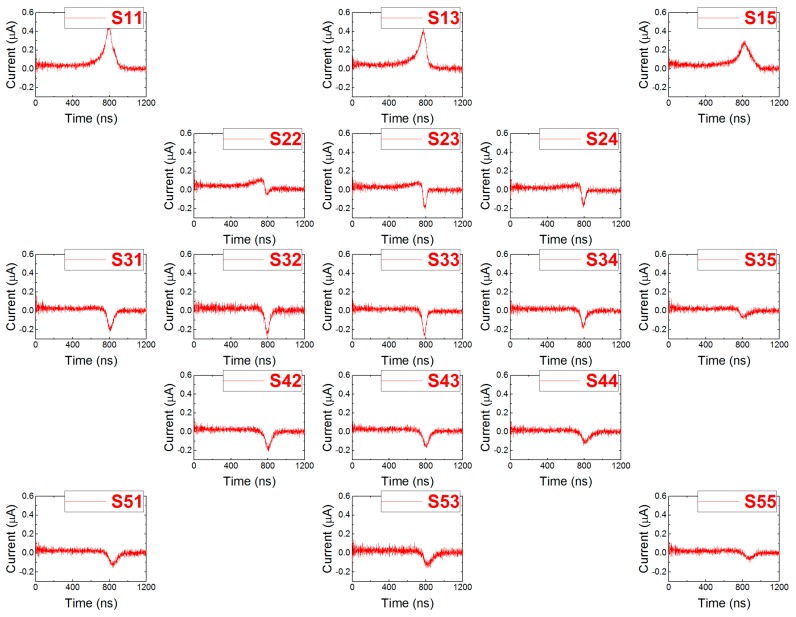
Current waveforms measured on GR at 400 V bias while illuminating different spots.

The collected charge (CC) on the electrode can be obtained from the respective L-TCT curves according to Equation (2). For the calculations, we used waveforms measured on CP and GR at 400 V bias, which are shown in Figure 9 and Figure 10, respectively. Each waveform was integrated to get the CC value. Finally, computed values were plotted into the contour maps using OriginPro 9 software.

**Figure 11:**
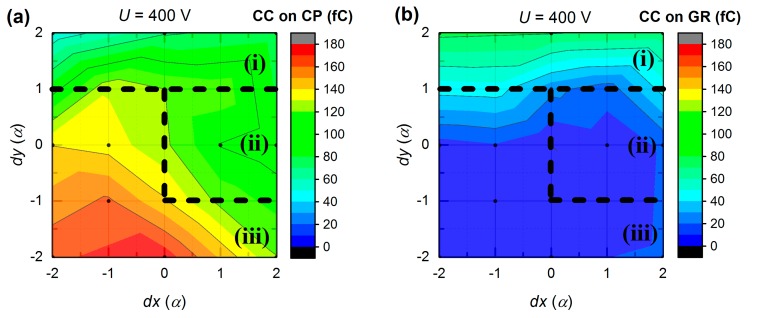
Dependence of collected charge (CC) on (**a**) CP and (**b**) GR on illumination position at 400 V bias (*α* = 0.85 mm).

Figure 11a presents the CC map around CP for current transients measured at 400 V bias. *dx* and *dy* axes denote the distance from S_33_ spot, where the step *α* = 0.85 mm. For example, the point at *dx* = −2*α*, *dy* = 0 corresponds to the value of CC on CP when illuminating S_31_. Likewise, the data for CC on GR is shown in Figure 11b. In both graphs, CC shows the values, which are not symmetrical to S_33_ point (*dx* = 0, *dy* = 0). Notably, CC on GR is significantly higher (40–80 fC) in the upper map part in Figure 11b, and remains almost zero (0–20 fC) elsewhere. At the same time, CC on CP is plateaued at 60–100 fC at the top and right part, and it is maximized at the bottom of Figure 11a. With the entire above mentioned, anode area can be divided into three regions of interest. In the region (i), the charge is collected on both electrodes, thus charge sharing occurs. In the region (ii), CC on CP is weak. Finally, in the region (iii), CP shows strong collection behavior.

**Figure 12:**
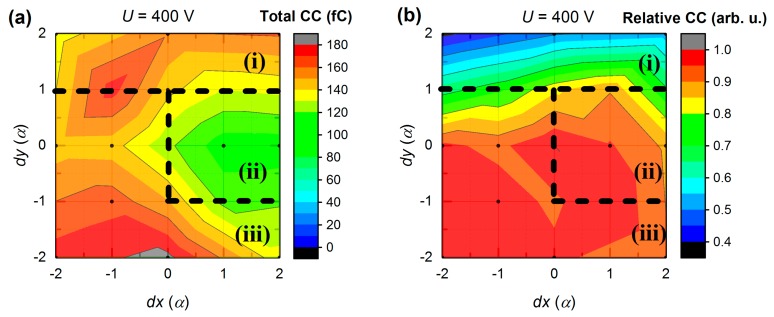
Dependence of (**a**) total CC and (**b**) relative CC on illumination position at 400 V bias (*α* = 0.85 mm).

The proposed region separation is further justified if we look at the total CC map shown in Figure 12a. As it is clearly seen, region (ii) possesses low CC values in contrast to other parts. In Figure 12b we also introduce relative CC:

(3) $$q_{relative}=Q_{CP}/Q_{total},$$

where *Q_CP_*—CC on CP, *Q_total_*—total CC at a given bias. This contour map is very similar to the one presented in Figure 11b due to the well-defined region (i), where the charge sharing occurs, and the relative CC varies rapidly with an illumination position. Thus, it may be possible to achieve the sub-pixel resolution in this area.

**Figure 13:**
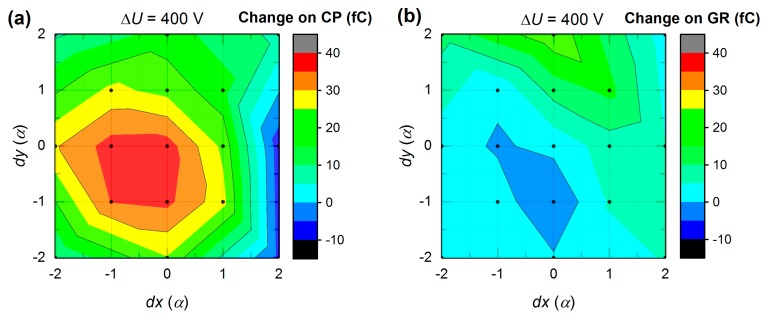
Maps of CC changes on (**a**) CP and (**b**) GR at Δ400 V bias variation (from 400 V to 800 V) (*α* = 0.85 mm).

Almost identical region patterns were obtained from L-TCT measurements at a higher bias (600 V and 800 V). While these direct results are omitted for simplicity, some additional information can be evaluated from bias-dependent CC changes. Figure 13a,b shows CC data calculated by subtracting CP and GR maps, respectively, obtained at 800 V (not shown) from the similar ones at 400 V (Figure 11) so that resulting maps show dependence of CC change on illumination position at 400 V bias variation (from 400 V to 800 V). It is seen in Figure 13a that the peak CC on CP gain (30–40 fC) is shifted from the CP center towards the bottom left corner and corresponds to previously defined region (iii). CC at the right side has zero to negative gain and partially lies within region (ii) boundaries. In Figure 13b, there is a negative valley that is broadly congruent with a maximum in Figure 13a. Those regions can be associated with increasing non-collecting behavior of GR and increasing charge collection on CP at higher biases, correspondingly.

**Figure 14:**
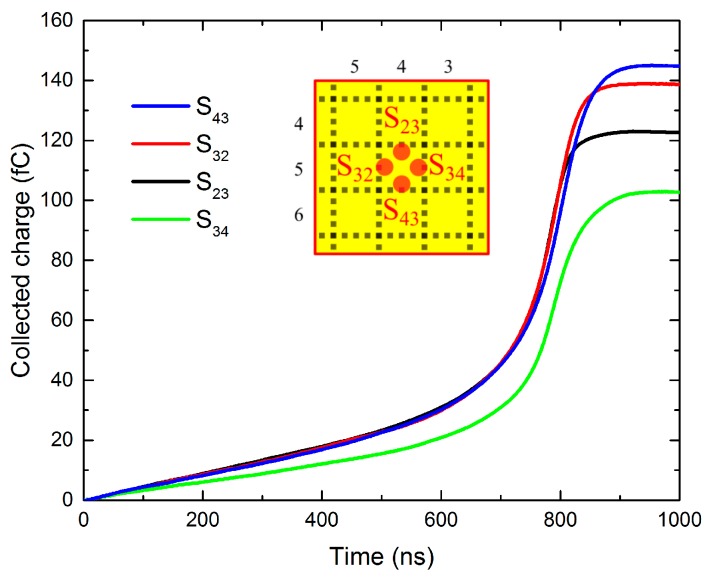
Temporal evolution of the charge collection in the spots distributed symmetrically around CP.

For further understanding of transient characteristics and associated region separation, the growth of the collected charge in time was investigated as well. It was calculated via the form

(4) $$Q(t)=\int_{0}^{t}I(t^{\prime })dt^{\prime },$$

where *Q*(*t*)—charge collected in time *t*, *I*(*t*’)—transient current dependent on time *t*’, integrated from 0 to *t*. For the analysis, we chose S_23_ (*dx* = 0, *dy* = 1), S_32_ (*dx* = −1, *dy* = 0), S_34_ (*dx* = 1, *dy* = 0), and S_43_ (*dx* = 0, *dy* = −1) illumination spots, which are symmetrically distributed around the CP, and 400 V bias CP data shown in Figure 9. Obtained curves are shown in Figure 14. As expected, S_32_ and S_43_ plots are similar, as they both belong to the region (iii) (see Figure 12). However, the charge collection on S_23_ shows noticeably lower profile after 800 ns while being similar to the previous plots elsewhere. This confirms the charge sharing effect in the region (i) (see Figure 12), which occurs due to the charge carriers splitting between CP and GR pixels adjacent to S_23_. The biggest depression at the collected charge is revealed at the S_34_ spot, where the signal is continuously dampened along the whole time scale. Simultaneously, the shape of the curve *Q*(*t*)*,* including characteristic collection time, remains nearly the same. This finding clearly proves that the main reason for the low CC in S_34_ and its surroundings is associated with a lower charge created by the photo-excitation near the cathode. Two principal reasons for this effect may be considered—(1) thicker gold contact inducing lower transmission, or (2) larger surface recombination in this region. This feature does not represent real drawbacks at X-ray detection. Since the CC remains low at the whole inspected bias range, the option (1) appears more probable.

In summary, we distinguished three different regions (see Figure 11 and Figure 12). Region (i) is characterized by significant charge sharing and is clearly separated in Figure 11b and Figure 12b. Region (ii) represents poor charge collection of the CP and is visible in Figure 12a. Low charge amount may be explained by the suppressed effectiveness of free charge generation by the laser light in this region. Region (iii) comprises the largest area and corresponds to the finest in terms of the performance part of the CP. In this case, the peak CP charge collection is expected to be in the position which overlaps with the peak from Figure 12a and the valley from Figure 12b. However, this spot is not centered on S_33_ (*dx* = 0, *dy* = 0) as it would be expected. Bolotnikov et al. [26] observed a phenomenon known as the “focusing” electric field associated with a positive space charge, which can be responsible for the observed shift of the CC peak.

## 4. Conclusions

We used the laser-induced transient current technique to study the propagation of the charge carriers through (CdZn)Te pixelated detector. Obtained current waveforms were correlated with the position of the illuminated spot on the cathode, the applied bias, and the charge collection on the specific electrodes: Central pixel and guard ring. We concluded that the analysis of the signal shapes might serve as a convenient tool for characterizing the pixel detector, especially concerning the character of the charge collection and the applicability of a detector for the subpixel resolution. The map of the collected charge was used to distinguish imperfect parts of the crystal in the studied area. Denoted regions were associated with charge sharing and contact imperfection, respectively, and were proven to have different impacts on the charge collection. Obtained maps of the collected charge in combination with current waveforms can be used at the identification of defective parts of the detector, as well as at the enhancement of its performance via numerical corrections optimized to the respective detector imperfections.

## Figures and Tables

**Table 1:** Position of illuminated spots. All measured distances have equal systematic fault ±0.25 mm.

Illuminated Spots	Distance FROM the Center of CP, mm
S_33_, S_CP_	0 ± 0.0005
S_23_, S_32_, S_34_, S_43_	0.85 ± 0.0005
S_22_, S_24_, S_42_, S_44_	1.2 ± 0.0005
S_13_, S_31_, S_35_, S_53_	1.7 ± 0.0005
S_11_, S_15_, S_51_, S_55_	2.4 ± 0.0005
S_AP_	2.55 ± 0.0005
